# Exploring the gap between hand washing knowledge and practices in Bangladesh: a cross-sectional comparative study

**DOI:** 10.1186/1471-2458-13-89

**Published:** 2013-01-30

**Authors:** Sifat E Rabbi, Nepal C Dey

**Affiliations:** 1 Research Associate, Environment Research Unit, Research and Evaluation Division, BRAC, BRAC Centre, 75, Mohakhali, Dhaka 1212, Bangladesh; 2 Research Fellow & Coordinator, Environment Research Unit, Research and Evaluation Division, BRAC, BRAC Centre, 75, Mohakhali, Dhaka 1212, Bangladesh

**Keywords:** Hand washing, Soap, Knowledge, Practices

## Abstract

**Background:**

Hand washing is considered as one of the most effective hygiene promotion activities for public health in developing countries. This study compared hand washing knowledge and practices in BRAC’s water; sanitation and hygiene (WASH) programme areas over time.

**Methods:**

This study is a cross-sectional comparative study between baseline (2006), midline (2009) and end-line (2011) surveys in 50 sub-districts from the first phase of the programme. Thirty thousand households from 50 sub-districts were selected in two steps: i) 30 villages were selected from each sub-district by cluster sampling, and ii) 20 households were chosen systematically from each village. The matched households were considered (26,404 in each survey) for analysis. Data were collected from households through face-to-face interview using a pre-tested questionnaire. Respondents were the adult female members of the same households, who had knowledge of day-to-day household activities related to water, sanitation and hygiene.

**Results:**

A gap between perception and practice of proper hand washing practices with soap was identified in the study areas. Hand washing practice with soap before eating was much lower than after defecation. In baseline data, 8% reported to wash their hands with soap which significantly increased to 22% in end line. Hand washing knowledge and practices before cooking food, before serving food and while handling babies is considerably limited than other critical times. A multivariate analysis shows that socio-economic factors including education of household head and respondent, water availability and access to media have strong positive association with hand washing with soap.

**Conclusion:**

Gap between knowledge and practice still persists in hand washing practices. Long term and extensive initiatives can aware people about the effectiveness of hand washing.

## Background

Hygiene promotions enhance the effectiveness of water and sanitation programme in most of the developing countries [1]. Hygienic behaviors can play an important role in the prevention of diseases related to water and sanitation. An average of 65% of death caused by diarrheal diseases could be reduced if good hygiene practice accompanies the provision of water and sanitation [2]. Diarrheal disease has been considered as a serious global problem [3] and leading cause of child mortality around the world [4]. Around 2.4 million deaths could be prevented annually by good hygiene practice, reliable sanitation and drinking water [5]. Evidence shows that hand washing can reduce the occurrence of diarrheal diseases by 14-40% [6]. Different studies showed that hand washing can decontaminate hands and prevent cross-transmission [7,8]. Hand washing with soap can also reduce the risk of endemic diarrhea and respiratory and skin infections [9]. The effectiveness of hand washing with soap can reduce diarrheal risk up to 47% [10]. Many studies carried out in Bangladesh suggested that hand washing is one of the factors which decreases the incidence of diarrhea in intervention areas [11,12]. Studies also revealed that WASH intervention improve the water, sanitation and hygiene situation in Bangladesh; reduce diarrheal prevalence associated with lower number of fecal-colony forming bacteria on hands [13].

Bangladesh faces many challenges related to water, sanitation and hygiene. Water and sanitation related disease is considered as one of the most significant child diseases in Bangladesh. Non-fatal chronic conditions such as diarrhea, worm infections, cholera, malaria, trachoma and schistosomiasis are also sourced from water and improper sanitation practice. Water-related diseases are responsible for 24% of all deaths and gastroenteritis and diarrheal diseases killing 110,000 children below the age of five every year in Bangladesh [14]. Improper sanitary practice such as open defecation, lack of proper hand washing practice, fecal disposal in open places are the major risk factors that results in diarrheal or water-borne diseases. Government of Bangladesh (GoB) has taken initiatives to achieve the full coverage of sanitation and in collaborating with BRAC (Bangladesh Rural Advancement Committee) WASH programme to achieve the target of Millennium Development Goal (MDGs).

The overall strategy of BRAC WASH programme is centered on hygiene and behavioral changes. In WASH intervention areas, BRAC form a Village WASH Committee (VWC) with 11 members (6 female and 5 male) from various groups (e.g. local elite, community people) for an average of 200 households. In each VWC, a total of 5–30 clusters were formed consisting of 10 households in each cluster. The programme assistant (PA) organize the cluster meetings ensuring the participation of female members from all 10 households at 2 months interval in each cluster. To ensure the active participation of male, adolescent and children (aged 9–11 years) in WASH activities, the Field Officer (FO) also organizes separate meetings apart from the cluster meetings. The programme also organizes different forums to ensure participation of local elites and other common people. The programme people disseminate knowledge of sanitation and hygienic practices through intensive health education. Besides the BRAC staff, the VWC members, religious leaders, community leaders, school student brigades also take part to implement the programme effectively at community and institutional levels.

To conduct the WASH initiatives in a new village, 3–4 days are needed to disseminate information about WASH and remind the community people of pervious BRAC interventions involving oral rehydration, drinking saline as health measure for diarrhea. BRAC WASH programme is the initial intervention that has undertaken to prevent the spread of diarrhea and other water borne diseases. After the dissemination, BRAC staff members extend an invitation for a cluster meeting in a selected house after consultation with the owner.

During the first day of the meeting, the staff members provide five messages on hand washing to make people aware about hygiene practices: three on hand washing before taking food and two on hand washing after defecation. Along with hygiene education, the health education component consists of awareness building on i) using safe water for cooking, washing and bathing; ii) keeping surroundings of the households, kitchen, tubewells and latrines tidy; iii) construct platform of the tubewells with solid materials; iv) disposal of domestic waste, excreta of poultry and livestock in fixed place and disposal of children’s feces in sanitary latrine. Then a map is drawn on the ground showing all households in the cluster. From that social mapping the availability and location of tubewell and sanitary latrines, water drainage system and the socio-economic system can be portrayed. BRAC-WASH staff members record the community people who have participated to draw the map and demonstrate the hand washing of those members. Then the next day, these people are divided into two groups that walk the whole area of the village and discussed what they have seen and what necessary steps are needed to take to ensure the safe water, sanitation and hygiene facilities for the village people. After the successful completion of the first meeting the committee members fix the place, time and date for the next meeting.

Before launching the WASH programme, a baseline survey was conducted by the Research and Evaluation Division in 2006–2007, to understand the pre-programme status vis-à-vis the impact evaluation of the programme. Subsequently, a midline survey was done during April-July, 2009 to assess the extent of changes occurred in different indicators including knowledge and practices in various intervention components. The aim of the end line survey (December 2009-March 2010) was to identify the impact of BRAC WASH programme on water, sanitation and hygiene practice after five years intervention period.

## Methods

### Study area and design

This is a cross-sectional comparative study of baseline, midline and end-line surveys. The surveys were carried out in 50 sub-districts where BRAC WASH programme has been offering its intervention since 2006.

### Sample size and sampling

The sample size comprised of 30,000 households, 600 from each of the study sub-districts for 3 rounds (baseline, midline and end-line surveys). A multi-stage sampling design was followed in drawing the sample. In the first step, using the 30 cluster sampling method, 30 villages were selected from each sub-district. In the second step, from each village, 20 households were selected systematically. A total of 29,985 households were interviewed at baseline (2006) while at midline (2009) the corresponding figure was 29,885 and end-line (2011) survey covered a total of 26,404 households. Only the matched households from the three consecutive years were considered for the comparison. The households which were missed in end-line survey (due to death, displacement and absenteeism) were not considered and thus the number of the households is smaller in the end-line than midline and baseline. Respondents were the adult female members of the same households, who had knowledge of day-to-day household activities related to water, sanitation and hygiene.

### Data management and analysis

Filled-in questionnaires were edited and coded for computer entry and twenty percent of the questionnaires were re-checked for consistencies. These data were entered in computer, cleaned and analyzed using the SPSS and STATA software (Licensed in 2007). Self-reported data were collected for hand washing practices in the three repeated survey period. Respondents were the adult female members of the same households, who had knowledge of day-to-day household activities related to water, sanitation and hygiene.The respondents were asked knowledge about the critical times of hand washing and how often they practice it. There were six critical times identified e.g. before eating, after defecation, after cleaning bottom of babies, before feeding babies, before cooking and before serving food. There were nine options for hand washing practices e.g. one hand with only water, two hands with only water, one hand with soap, two hands with soap, one hand with soil, two hands with soil, one hand with ash, two hands with ash and do not wash. It was considered that they would say those critical periods when they practice hand washing and how they practice in those critical periods usually. If any respondent failed to notice any critical time of hand washing then it was considered as ‘do not wash hands’ for that critical time. In case, absence of the baseline’s respondent in midline or in end line survey, any adult household member was interviewed from those particular households. Chi-square test compared the significance of differences between indicator values. A ‘*p’ value* of 0.05 or less was considered as significant chi-square tests.

### Statistical analysis

#### Index for hand washing at critical time

Generalized Linear Model (GLM) was used to analyze factors associated with hand washing practices. We detailed out the specific indicators used to construct the index. For constructing the index, we assigned a value of ‘1’ for highest score of self-reported hand washing practice of both hands with soap at critical times whereas ‘0’ for lowest score when none washed hands at all. Then the values were summed up and divided by total number of indicators used so that is the value of the index lies between ‘0 to 1’. This index were used as dependent variable for the regression analysis to see what or which factors influence hygienic hand washing practices at critical times. Thus, we created a variable named ‘Wash score’ for multivariate analysis. Six critical times for hand washing are considered while constructing the score, e.g. before taking meal, before serving foods, before cooking foods, after defecation, before feeding babies and after cleaning bottom of babies.

### Ethical issues

To conduct the study ethical clearance was obtained from BRAC’s own ethics committee at Research and Evaluation Division (RED). Verbal consent was obtained from the participants before conducting interviews. The enumerators read out the verbal consent form to inform the general objectives of the study to the participants. They were also informed that their participation was completely voluntary and could withdraw from the interview at any time. The respondents were also free to refuse answer to any questions they felt uncomfortable. Any refusal would not affect receiving any services from BRAC. Confidentiality in data handling was strictly maintained.

## Results

### Gap between knowledge and practice of hand washing with soap

**Figure 1:**
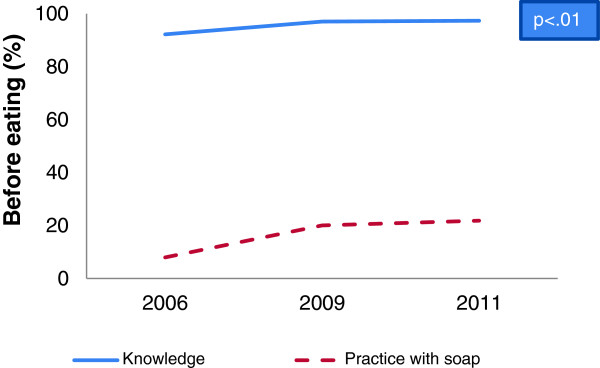
Knowledge and practice gap of hand washing before eating.

**Figure 2:**
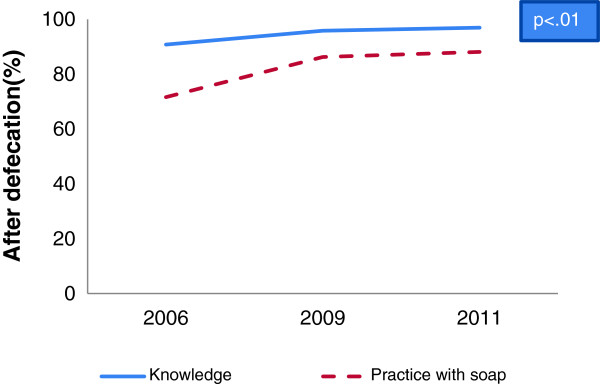
Knowledge and practice gap of hand washing after defecation.

**Figure 3:**
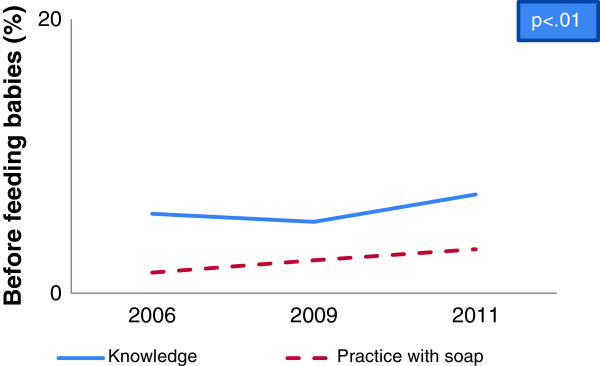
Knowledge and practice gap of hand washing before feeding babies.

**Figure 4:**
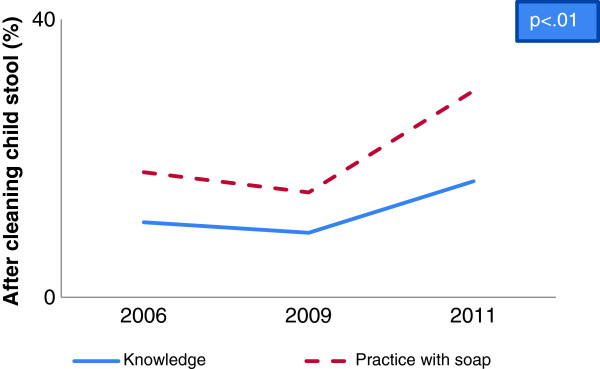
Knowledge and practice gap of hand washing after cleaning child’s stool.

A gap between hygienic hand washing knowledge and self-reported practices was identified in baseline, midline and end-line data. Overall 95% respondents reported that hand washing is essential before taking food whereas 8% of them wash their hands with soap in baseline which increased in midline to 20% and in end-line to 22% (Figure 1). Analysis also revealed that hand washing practice with soap after defecation increased from baseline to midline as well as end-line (72% vs. 86% vs. 88%) whereas more than 90% respondents reported that they had knowledge (Figure 2). Besides, hand washing knowledge before serving food and practice with soap increased significantly from baseline to midline and end-line (1% vs. 2% vs. 3.6%) (Table 1 and Table 2). On the other hand, a reverse situation was found in the case of after cleaning child’s stool where practice was found higher than knowledge. In some cases, depletion of knowledge (5.8% vs. 5.2%) was found from baseline to midline for before feeding babies (Figure 3) and midline to end-line for before cooking food (44% vs. 40%) (Table 1). But hand washing practice with soap increased significantly from baseline to midline and to end-line before cooking food (Table 2) and before feeding babies (Table 3). In the end-line, 30% respondents reported they practiced hand washing with soap after cleaning bottom of babies, whereas 17% reported it as critical time (Figure 4). Hand washing with only water extensively high before eating in baseline (84%) which decreased in end-line (75%) (Table 2). A major percentage of respondents reported that they did not wash hands before serving food and before cooking across the survey periods (Table 2) and knowledge regarding these issues were also insufficient (Table 1).

**Table 1:** Self-reported knowledge about the critical time of hand washing (%)

**Critical times**	**Survey year**						**Relative difference and p value**		
	**2006**		**2009**		**2011**		**2006-2009**	**2009-2011**	**2006-2011**
	**Yes**	**No**	**Yes**	**No**	**Yes**	**No**			
Before taking meal	92.2	7.8	97.0	3.0	97.3	2.7	5.21 (P<0.01)	0.31 (ns)	5.1 (P<0.01)
Before serving food	10.8	89.2	11.3	88.7	13.6	86.4	4.63 (ns)	20.35 (P<0.01)	2.8 (P<0.01)
Before cooking	30.4	69.6	44.3	55.7	40.0	60.0	45.72 (P<0.01)	−9.71 (P<0.01)	10.4 (P<0.01)
After defecation	90.8	9.2	95.8	4.2	96.9	3.1	5.51 (P<0.01)	1.15 (P<0.01)	6.1 (P<0.01)
Before feeding babies	5.8	94.2	5.2	94.8	7.2	92.8	−10.34 (ns)	38.46 (P<0.01)	1.4 (P<0.01)
After cleaning child’s stool	10.8	89.2	9.3	90.7	16.7	83.3	−13.89 (P<0.01)	79.57 (P<0.01)	6.0 (P<0.01)
N	26,404								

**Table 2:** Self-reported hand washing (one/both hands) practice with soap (%)

**Wash hands**	**Survey year**			**Relative difference and p value**		
	**2006**	**2009**	**2011**	**2006-2009**	**2009-2011**	**2006-2011**
**Before taking food**						
Soap	7.9	20.0	21.8	153.16 (p<0.01)	1.8 (p<0.01)	13.8(p<0.01)
Water	84.3	77.0	75.5	−8.66 (p<0.01)	−1.5 (p<0.01)	−8.8(p<0.01)
Don’t wash	7.8	3.0	2.7			
**Before serving food**						
Soap	1.0	1.9	3.6	90.0 (p<0.01)	89.47 (ns)	260 (p<0.01)
Water	9.8	9.4	10.0	−4.08 (ns)	6.38 (ns)	2.04 (ns)
Don’t wash	89.2	88.7	86.4			
**Before cooking**						
Soap	3.3	7.1	9.1	115.15 (p<0.01)	28.17 (p<0.01)	175.76 (p<0.01)
Water	27.1	37.1	30.8	36.90 (p<0.01)	−17 (p<0.01)	13.65 (p<0.01)
Don’t wash	69.6	55.7	60.0			
**After defecation**						
Soap	71.6	86.2	88.1	20.39 (p<0.01)	2.04 (p<0.01)	23.04 (p<0.01)
Water	19.2	9.6	8.8	−50.0 (p<0.01)	−8.33 (p<0.01)	−54.17 (p<0.01)
Don’t wash	9.2	4.2	3.1			
N	26,404					

**Table 3:** Self-reported hand washing (one/both hands) practice with soap while handling babies (%)

**Wash hands**	**Survey year**			**Relative difference and p value**		
	**2006**	**2009**	**2011**	**2006-2009**	**2009-2011**	**2006-2011**
**Before feeding babies**						
Soap	1.5	2.4	3.2	60 (p<0.01)	33.33 (p<0.01)	113.33 (p<0.01)
Water	8.2	5.7	7.9	−30.48 (p<0.01)	38.60 (p<0.01)	−3.66 (ns)
Don’t wash	90.2	91.8	88.7			
**After cleaning child’s stool**						
Soap	18	15.1	29.7	−16.11 (p<0.01)	96.69 (p<0.01)	12 (p<0.01)
Water	8.6	4.4	7.6	−48.83 (p<0.01)	72.73 (p<0.01)	−11.63 (p<0.01)
Don’t wash	73.3	80.4	62.6			
N	9108	11345	7473			

### Factors associated hand washing practice

The association between hand washing practice and different socio-economic indicators is shown (Table 4). Hand washing practice increased significantly from baseline to midline and end-line (*p*<0.01). Among the independent variables education have strong association with hand washing practices. Both household head’s education and spouse’s education have significant relation with education (*p*<0.01). Access to media (television) is significantly associated with hand washing practice whereas electricity has no significant impact on hand washing (*p*<0.01). Hand washing practice was significantly associated with tubewell and latrine ownership (*p*<0.01). Besides, hand washing materials (e.g. soap, ash) and water availability near latrines also have significant positive association with hygienic hand washing practices. NGO membership including BRAC has strong association with hand washing practices.

**Table 4:** Results of GLM estimates for the factors influencing hand washing practices

**Indicators**	**Dependent value: Hand washing co-efficient**	**z value**	**95% conf. interval**
**Survey year**			
Year 2009 (1 if year 2009, 0 if year 2006 and 2011)	0.060	59.47***	0.0577-0.061
Year 2011 (1 if year 2011, 0 if year 2006 and 2009)	0.075	67.22***	0.0726-0.077
**Education**			
Education of household heads (years)	0.002	15.56***	0.001-0.002
Education of respondents (years)	0.003	21.03***	0.002-0.003
**Occupation**			
Occupation of HH (1=service, 0=others)	0.004	1.93*	−0.000-0.007
Occupation of HH (1=business, 0=others)	0.001	1	−0.001-0.003
Occupation of HH (1=farming, 0=others)	0.000	0.61	−0.001-0.002
**Household assets**			
Electricity (1 if Yes, 0 if No)	0.000	0.85	−0.001-0.002
Television (1 if Yes, 0 if No)	0.013	11.49***	0.010-0.015
Roof material (1if concrete, 0 if others)	0.002	0.82	−0.003-0.008
Floor material (1if concrete, 0 if others)	0.008	4.49***	0.004-0.117
Wall material (1if concrete, 0 if others)	−0.000	−0.12	−0.002-0.002
**Economic status**			
Perceived economic status (1 if deficit and 0 if surplus)	−0.008	−9.09***	−0.009-(−0.006)
**Others**			
Tubewell ownership (1if yes, 0 if No)	0.0186	16.13***	0.016-0.021
Latrine ownership (1 if yes, 0 if No)	0.016	17.29***	0.014-0.018
Water availability near latrine (1 if Yes, 0 if No)	0.003	2.62**	0.000-0.004
Soap/ash near latrine (1 if Yes, 0 if No)	0.022	16.13***	0.019-0.025
Slipper near latrine (1 if Yes, 0 if No)	0.028	15.2***	0.024-0.032
**Membership**			
BRAC member (1 if BRAC members, 0 if others)	0.008	6.49***	0.006-0.011
Other NGO member (1 if other NGO Member, 0 if BRAC member)	0.005	6.75***	0.004-0.007
Constant	0.177	115.19***	0.174-0.180

Note: ***, ** and * denote significant level at 1%, 5% and 10%, respectively.

## Discussion

This study has identified gap between hand washing knowledge and practice with soap at different critical times i.e. before eating, after defecation, after cleaning child’s stool, before feeding babies and before cooking and serving food across programme intervention period. Analysis further revealed that majority (90%) of respondents have knowledge about hand washing with soap before eating and after defecation, but only 21% and 88% respondents reported to do so respectively. This finding illustrates the knowledge-behavior gap in hand washing with soap. A recent study carried out in Kenya supports our study findings where 71% respondents understood the importance of hand washing after defecation but only 31% did so [15]. Improvement status of hand washing with soap after defecation was found in end-line than baseline and midline. It was also found that reported hand washing practice with soap before eating is much lower than after defecation. A study conducted by Shabnam (2010) in Jamalpur district in Bangladesh reports similar findings regarding hand washing. Furthermore, results showed that reported hand washing practice with soap after defecation is much higher than actual practice in Bangladesh [16,17]. The findings demonstrate that verbal response about hand washing behavior does not provide a real scenario of practices. Hand washing with only water before eating was higher during the consecutive survey period as it seemed sufficient cleanliness for them. Similar finding was found in the study by Halder et al. 2010 [18]. Besides, most of the respondents did not mention about hand washing before cooking and before serving food.

The respondents having children (<5 years) reported hand washing practice with soap at two times, e.g. before feeding and after cleaning child’s stool. Responses’ regarding hand washing before feeding babies indicate this practice is not common and requires greater emphasis to develop practice in future. Another observational study on hand washing also indicated the similar findings (Halder et al. 2010). Findings also revealed that hand washing knowledge after cleaning bottom of babies is lower than practice. It can be assumed that respondents from those households do not have children failed to point out this critical time of hand washing and practice. Besides, knowledge depletion was also found from baseline to midline before feeding babies and after cleaning child’s stool. It might happen in some cases, as alternative respondents were chosen during the repeated survey periods from the same household due to the absence of previous respondent. Moreover, this is also likely due to the lack of proper intervention and monitoring facilities after three years of programme intervention. After the midline evaluation the programme again gave more emphasis on hygiene education and hand washing knowledge which focused in the end line evaluation. Whereas, knowledge and practice regarding hand washing with soap after cleaning bottom of babies, has been increasing significantly from baseline to end-line.

In the multivariate analysis, hand washing practice is strongly influenced by indicators such as education, media access, better economic condition, etc. The education of household head and respondent has greater emphasis on hygienic hand washing practice. Hygiene practice regarding hand washing increases if the level of education increases. Besides, media access (television) has strong association in the practice of hand washing rather than access to electricity. In addition, being financially better off have positive influence on hand washing practices. Moreover, some relevant issues related to hand washing, such as, latrine ownership, tubewell ownership, water/soap/slipper availability near latrine are also associated. It is assumed that if water, soap and slippers are available near latrines then people would be more conscious about hygienic hand washing practice. In addition, different NGO memberships including BRAC have strong association in hand washing practices.

Although this study endures a methodological weakness because of the absence of control group. In addition, hand washing practice with soap at different critical times was not physically verified which is also a limitation of this study. Structured observation is imperative to assess the real scenario of hygiene practices. The strength of the study including separate teams of field investigators for data collection during baseline, midline and end-line, analysis of data of the same households across the surveys might help avert information bias.

## Conclusion

Understanding the changing behaviour of hand washing in the intervention areas was the major concern of this study. Gap between knowledge and practice of hand washing with soap persist and long term motivating activities are needed to improve hand washing practice with soap. In some critical period of hand washing, the community people are still unconscious about the necessity of hand washing with soap. But, the scenario has been changing and the percentage of people who wash their hands with soap has been increasing during the intervention period. Long term and extensive initiatives should be under taken to make people aware about the effectiveness of hand washing.

## Competing interest

The authors declare that they have no competing interests.

## Authors’ contribution

Conception, design, analysis and interpretation of data and writing: SER; Involved in drafting the manuscript: NCD. Authors read and approved the final manuscript.

## Pre-publication history

The pre-publication history for this paper can be accessed here:

http://www.biomedcentral.com/1471-2458/13/89/prepub

## References

[B1] Sijbesma C Christoffers T The value of hygiene promotion: cost-effectiveness analysis of interventions in developing countries Health Policy Plann 2009 24 6 418 427 10.1093/heapol/czp036 19703917

[B2] WaterAid. Hygiene 2009 http://www.wateraid.org/uk/what_we_do/the_need/5901.asp (accessed on 12 January 2011)

[B3] WHO Health Statistics Mortality and burden of diseases 2008 Geneva, Switzerland: WHO http://www.who.int/whosis/whostat/EN_WHS08_Table1_Mort.pdf

[B4] Boschi-Pinto C Velebit L Shibuya K Estimating child mortality due to diarrhea in developing countries Bull World Health Organ 2008 86 710 717 10.2471/BLT.07.050054 18797647 PMC2649491

[B5] Prüss-Üstün A Bos R Gore F Bartram J Safer water, better health: costs, benefits and sustainability of intervention to protect and promote health 2008 Geneva: World Health Organization

[B6] Hoque BA Hand washing practices and challenges in Bangladesh Int J Environ Heal R 2003 13 1 81 87 10.1080/0960312021000071567 12775383

[B7] Kaltenthaler E Waterman R Cross P Faecal indicator bacteria on the hands and the effectiveness of hand-washing in Zimbabwe J Trop Med Hygiene 1991 1991 94 5 358 63 1942217

[B8] Larson EL APIC guideline for handwashing and hand antisepsis in health care settings Am J Infect Control 1995 23 4 251 69 10.1016/0196-6553(95)90070-5 7503437

[B9] Bartram J Cairncross S Hygiene, Sanitation and Water: Forgotten foundations of Health PLoS Med 2010 7 11 1000367 10.1371/journal/pmed.1000367 PMC2976722 21085694

[B10] Curtis V Cairncross S Effect of washing hands with soap on diarrhoea risk in the community: a systematic review Lancet Infectious Diseases 2003 3 5 275 81 10.1016/S1473-3099(03)00606-6 12726975

[B11] Stanton BF Clemens JD An educational intervention for altering water-sanitation behaviors to reduce childhood diarrhea in urban Bangladesh. II. A randomized trial to assess the impact of the intervention on hygienic behaviors and rates of diarrhea. Am J Epidemio 1987 125 292 301 10.1093/oxfordjournals.aje.a114529 3812435

[B12] Alam N Henry FJ Rahaman MM Wojtyniak B Mother’s personal and domestic hygiene and diarrhoea incidence in young children in rural Bangladesh Int J Epidemio 1989 18 242 247 10.1093/ije/18.1.242 2722372

[B13] Hoque BA Juncker T Sack RB Ali M Aziz KMA Sustainability of a water, sanitation and hygiene education project in rural Bangladesh: a 5-year follow-up Bull WHO 1996 74 431 437 8823966 PMC2486877

[B14] WaterAid 2011 http://www.wateraid.org/uk/what_we_do/where_we_work/bangladesh/ (accessed on 10 December 2011)

[B15] Yolande C Jacqueline D Introducing FOAM 2010 A framework to analyze handwashing behaviours to design effective handwashing programs, working paper of Water and Sanitation program

[B16] ICDDR B Post defecation hand washing in Bangladesh 2008 Dhaka, Bangladesh: Practice and efficiency perspective

[B17] Shabnam L The practice of hand washing 2010 Dhaka, Bangladesh: South Asia Hygiene Practitioners Workshop

[B18] Halder A Carole T Akhter S Bhuiya A Johnstone R Luby S Observed hand cleanliness and others measures of hand washing behaviors in rural Bangladesh BMC Public Health 2010 10 545 10.1186/1471-2458-10-545 20828412 PMC2944374

